# Genetic and Phenotypic Analysis of Phage-Resistant Mutant Fitness Triggered by Phage–Host Interactions

**DOI:** 10.3390/ijms242115594

**Published:** 2023-10-26

**Authors:** Yanze Mi, Yile He, Jinhui Mi, Yunfei Huang, Huahao Fan, Lihua Song, Xiaoping An, Shan Xu, Mengzhe Li, Yigang Tong

**Affiliations:** 1 College of Life Science and Technology, Beijing University of Chemical Technology, Beijing 100029, China; 2021210737@buct.edu.cn (Y.M.); hyl06991201@163.com (Y.H.); mijinhuinl@163.com (J.M.); huangyunfei2000@163.com (Y.H.); fanhuahao@mail.buct.edu.cn (H.F.); songlihua@mail.buct.edu.cn (L.S.); ananxiaoping@sina.com (X.A.); shanxu@buct.edu.cn (S.X.); 2 Beijing Advanced Innovation Center for Soft Matter Science and Engineering (BAIC-SM), Beijing University of Chemical Technology, Beijing 100029, China

**Keywords:** phage resistance, fitness cost, phage steering, phage therapy, receptor-related mutants

## Abstract

The emergence of phage-resistant bacterial strains is one of the biggest challenges for phage therapy. However, the emerging phage-resistant bacteria are often accompanied by adaptive trade-offs, which supports a therapeutic strategy called “phage steering”. The key to phage steering is to guide the bacterial population toward an evolutionary direction that is favorable for treatment. Thus, it is important to systematically investigate the impacts of phages targeting different bacterial receptors on the fitness of the bacterial population. Herein, we employed 20 different phages to impose strong evolutionary pressure on the host *Pseudomonas aeruginosa* PAO1 and examined the genetic and phenotypic responses of their phage-resistant mutants. Among these strains with impaired adsorptions, four types of mutations associated with bacterial receptors were identified, namely, lipopolysaccharides (LPSs), type IV pili (T4Ps), outer membrane proteins (OMPs), and exopolysaccharides (EPSs). PAO1, responding to LPS- and EPS-dependent phage infections, mostly showed significant growth impairment and virulence attenuation. Most mutants with T4P-related mutations exhibited a significant decrease in motility and biofilm formation ability, while the mutants with OMP-related mutations required the lowest fitness cost out of the bacterial populations. Apart from fitness costs, PAO1 strains might lose their resistance to antibiotics when counteracting with phages, such as the presence of large-fragment mutants in this study, which may inspire the usage of phage–antibiotic combination strategies. This work provides methods that leverage the merits of phage resistance relative to obtaining therapeutically beneficial outcomes with respect to phage-steering strategies.

## 1. Introduction

Antibiotics are essential for combating bacterial infections. However, their efficacy is imperiled by the emergence of multidrug-resistant bacteria in clinical settings due to their overuse and misuse. The overuse of antibiotics has made *Pseudomonas aeruginosa* one of the most common multidrug-resistant bacteria, and it is resistant to a wide range of antibiotics, including beta-lactams, fluoroquinolones, and aminoglycosides [1]. In addition, the discovery and development of new antibiotics are becoming increasingly challenging due to the high cost and slow pace. This indicates that antibiotic resistance is a growing global public health threat, driving the exploration of alternative therapies relative to antibiotics in medicine [2]. As a result, alternative treatments are needed, and phage therapy offers a potential treatment for highly drug-resistant infections. Compared with antibiotics, phage therapy offers several advantages, including high specificity, minimal adverse effects, low developmental costs, and ease of modification [3]. At present, phage therapy has become a multifaceted and comprehensive approach, and it is not limited to the traditional antimicrobial strategy, which is based on lytic phages or phage cocktails [4], but rather encompasses a range of therapeutic techniques. Phage therapy includes phage–antibiotic combinations [5], phage-derived enzymes [6], phage steering based on bacterial fitness trade-offs due to phage resistance [7], and antibiotic sensitivity restoration mediated by temperate phages [8].

One of the challenges in the clinical application of phage therapy is the emergence of phage resistance in bacteria [9]. Bacteria and phages engage in an arms race due to continuous exposure to each other, resulting in high mutation rates and horizontal gene transfer, which enhance their genetic traits and diversity [10]. Among the various defense mechanisms against phages, changes in bacterial receptors due to mutations in the genome may be the simplest way for bacteria to acquire phage resistance.

Gram-negative bacteria use different receptors to interact with phages, such as lipopolysaccharides (LPSs), type IV pili (T4Ps), outer membrane proteins (OMPs), and exopolysaccharides (EPSs) [11]. These receptors also have other functions, such as surface antigens, virulence factors, nutritional auxiliary factors, and effluent carriers [12,13]. Accordingly, such bacterial receptor-associated mutations may cause bacteria to develop trade-off strategies with respect to adaptive costs, such as lowered fitness [14], reduced virulence [15], and decreased antibiotic resistance [16]. Consequently, Gurney et al. proposed the concept of “phage steering”, which uses the specific phage resistance of pathogenic bacteria to improve infection outcomes [17]. In this concept, the bacteria surviving after phage infections will be phage-resistant, but they will exhibit reduced virulence relative to the hosts or become more sensitive to antibiotics. This approach can direct bacterial populations toward a more favorable therapeutic direction by using different phages or phage cocktails, enabling increased scientific phage therapy during clinical treatment. Phage steering is a novel therapeutic strategy that exploits the trade-offs between phage resistance and bacterial fitness.

*Pseudomonas aeruginosa* (*P. aeruginosa*) is a Gram-negative opportunistic pathogen that can resist most antibiotics via intrinsic and acquired mechanisms [18], such as beta-lactams, fluoroquinolones, and aminoglycosides [1]. It is considered a top-priority pathogen that requires immediate attention and alternative treatments, such as phage therapy or phage steering. However, the effects of phage-resistant mutations on bacterial fitness traits, such as growth rates, motility, biofilm formation, virulence factors, and antibiotic resistance, have not been systematically investigated in *P. aeruginosa* strains. In this work, using *P. aeruginosa* PAO1 as an example, we harnessed 20 different phages that can infect PAO1 and impose strong evolutionary selection on the host strain, and we systematically examined the impacts of PAO1 resistance relative to different phages at both the genotype and phenotype levels. Our work can provide a basis for understanding the fitness of phage-resistant bacteria and their relationship with the bacterial receptors of phages. The aim of our study is to provide guidelines for selecting appropriate phages or phage cocktails in order to guide phage-resistant bacterial populations toward a favorable direction for phage steering; these guidelines focus on the following: reduced fitness, motility, biofilm formation, and virulence, and increased antibiotic susceptibility.

## 2. Results

**Figure 1:**
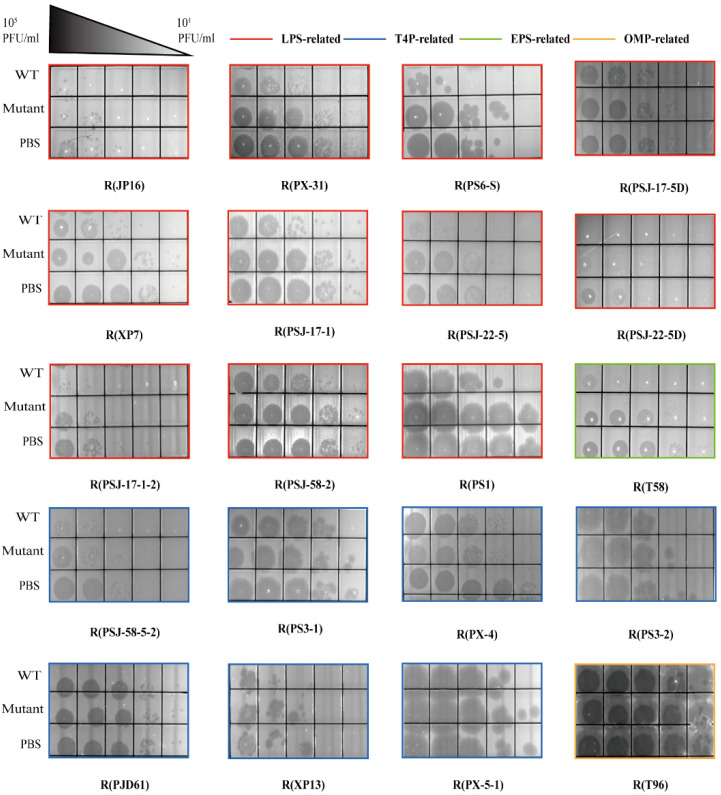
Adsorption estimation of different phages on PAO1 and its mutants. Different colors of the frames indicate the mutated genes related to different bacterial receptors.

**Figure 2:**
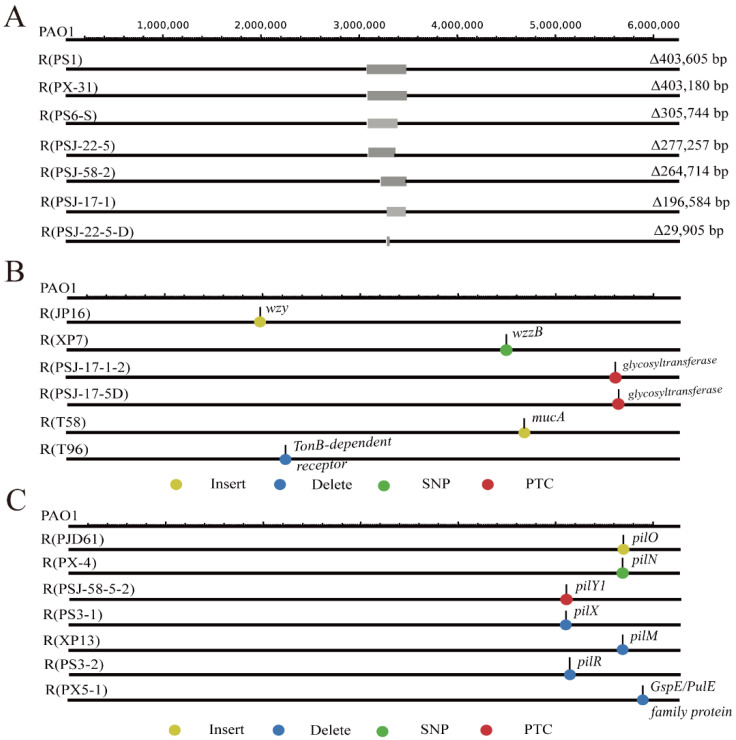
A graphical representation of the genome-wide mutation that occurred in PAO1 mutants with impaired phage adsorption. (**A**) LPS-related large-fragment deletion mutants; (**B**) other LPS-related, OMP-related, EPS-related mutants; (**C**) T4P-related mutants. The color dots refer to the different mutation types, and the gray bands indicate the large-fragment deletion (SNP: single-nucleotide polymorphism; PTC: premature termination codon).

The phenotypic and genotypic characterization of phage-resistant PAO1 strains: A total of 20 phages infecting PAO1 (Table S1) were used to impose evolutionary selection on the host strain, and we observed the emergence of 20 PAO1 mutants that could survive after the corresponding phage infection and investigated their phenotypic and genotypic characteristics. On the one hand, we adopted an adsorption assay to confirm the phage-resistant phenotype. Based on the classification of surface receptors, we divided the mutations into four groups, namely, LPS-related genes, T4P-related genes, OMP-related genes, and EPS-related genes. As shown in Figure 1, all phages could adsorb to wild-type PAO1, and there was no phage adsorption or impaired adsorption for the mutants. On the other hand, we analyzed the genotypic information of all phage-resistant PAO1 strains by comparing it with that of the wild-type PAO1 (Table S1). The mutations were mapped in the reference PAO1 genome (Figure 2), which included insertion, deletion, and substitution. A comparative analysis showed the presence of LPS-related gene mutations in 11 out of 20 phage-resistant PAO1 strains, including large-fragment deletions (7 cases, Figure 2A), single-nucleotide polymorphism (SNP, 1 case of SNP in *wzzB* and 2 cases of SNP in glycosyltransferase, Figure 2B), and insertion (1 case, Figure 2B). Among these mutants, the deletion sizes of the seven mutants with large-fragment deletions ranged from 29,905 bp to 403,605 bp, all of which included the *galU* gene. This gene encodes UTP-glucose-1-phosphate uridylyltransferase, which relates to the synthesis of LPS. With the *hmgA* gene included in the large-fragment deletions, all large-fragment deletion strains, except for R (PSJ-22-5D), had a brown phenotype. In addition, other LPS-related mutants showed the presence of mutations in three genes, namely, the LPS O-antigen chain length determinant protein gene *wzzB*, the O-antigen polymerase gene *wzy*, and the glycosyltransferase gene. The genome sequences of wild-type PAO1 and mutants were deposited at the National Centre for Biotechnology Information (NCBI), under the BioProject accession number PRJNA963233.

### 2.1. Growth Curves

**Figure 3:**
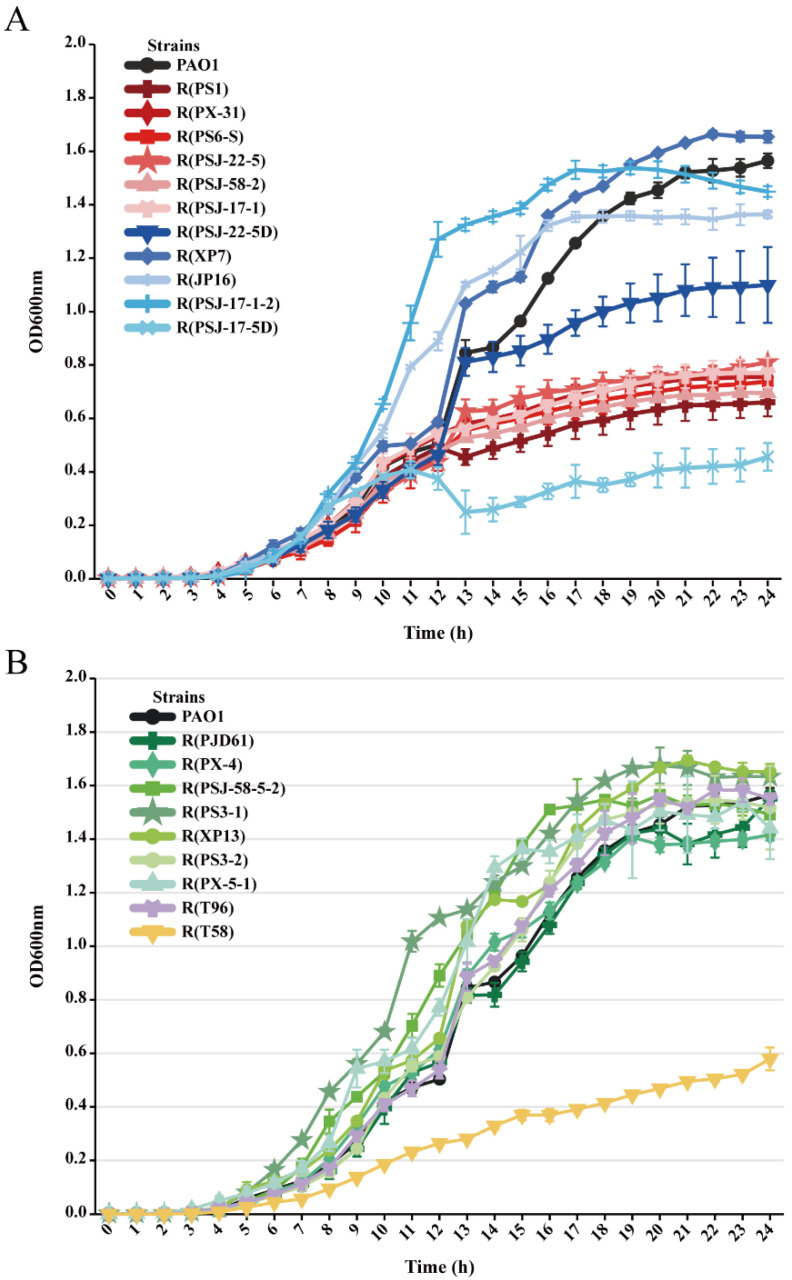
Growth curves of different mutant populations as indicators of bacterial fitness, expressed as OD_600_ values of 24 h incubation at 37 °C measured hourly. (**A**) LPS-related mutants; (**B**) T4P-related and other related mutants.

As shown in Figure 3A, the large-fragment deletion mutants showed significantly high fitness costs in growth compared to the wild-type PAO1 (*p* < 0.05). The maximum OD of the strains with the brown phenotype decreased by about 50% compared with that of the wild-type PAO1 after 24 h incubation. R(PSJ-22-5D), with only a 29,905 bp deletion, showed less impaired growth than the strains with the brown phenotype, and its maximum OD was reduced by about 25% compared with that of the wild-type PAO1. Additionally, other LPS-related mutants showed varying degrees of growth changes. R(XP7) showed growth advantages and an increase in maximum OD, whereas R(JP16) exhibited growth advantages initially but a reduction in maximum OD after 24 h of incubation. The fitness costs caused by mutations were not only related to specific genes but also strongly influenced by the mutation site. In the case of premature termination codon (PTC)-type mutations, compared to R(PSJ-17-1-2), R(PSJ-17-5D) showed a more significant decrease in maximum OD and fluctuation in growth. Unlike LPS-related mutants, the growth of all T4P-related mutant strains and the OMP mutant strain R(T96) did not differ significantly (*p* > 0.05) when compared to that of the wild-type PAO1 (Figure 3B). Some of the T4P-related mutants, such as R(PS3-1) and R(PSJ-58-5-2), had a slightly higher maximum OD than the wild-type PAO1. However, the EPS-related mutant strain R(T58) showed the highest fitness cost among all phage-resistant strains in terms of growth, and it did not display a discernible logarithmic phase, suggesting a possible defect in its growth kinetics.

### 2.2. Motility Assays

**Figure 4:**
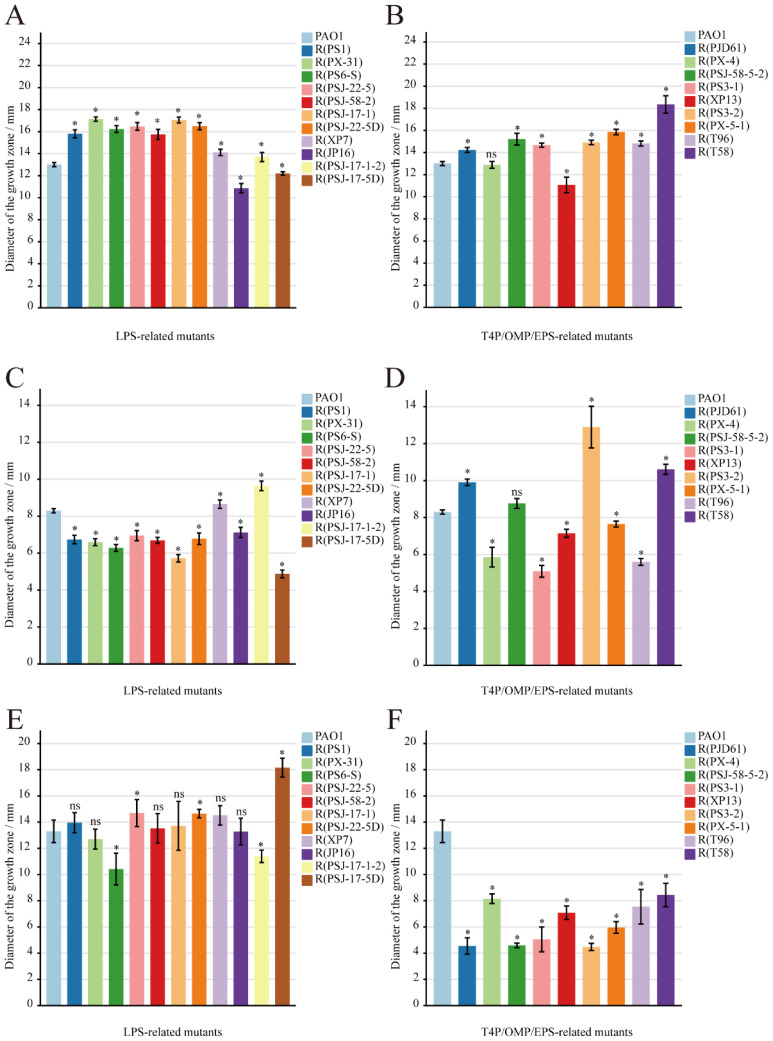
Three motility assessments of PAO1 and its mutants, expressed as the diameter of the growth zone. (**A**) LPS-related mutants, swimming; (**B**) T4P-related and other related mutants, swimming; (**C**) LPS-related mutants, swarming; (**D**) T4P-related and other related mutants, swarming; (**E**) LPS-related mutants, twitching; (**F**) T4P-related and other related mutants, twitching. Symbol * indicates the sample is significantly different (*p* < 0.05) from the control wild-type PAO1, and “ns” means no significance.

We measured three motilities using Luria–Bertani (LB) agar plates with different agarose concentrations (0.3% for swimming, 0.5% for swarming, and 1.0% for twitching) and assessed the strength of these motilities via the diameter of the growth zone (Table S2). In Figure 4A, most of the LPS-related mutant strains showed increased swimming motility when compared to the wild-type PAO1 (13.0 ± 0.2 mm), except for R(JP16) and R(PSJ-17-5D). It is worth noting that all strains with the brown phenotype showed a significant increase (*p* < 0.05) in swimming motility (>15 mm). In addition, the swimming motility of the phage-resistant strains with other mutations also varied significantly in comparison with that of the wild-type PAO1 (*p* < 0.05). As shown in Figure 4B, 75% of T4P-related mutants showed enhanced swimming motility, and the EPS-related mutant R(T58) exhibited the strongest swimming motility among the mutants (18.4 ± 0.8 mm). Figure 4C reveals that there was a significant (*p* < 0.05) decrease in most LPS-related mutants’ swarming motility (<8.3 ± 0.1 mm), except for R(PSJ-17-1-2) and R(XP7), which exhibited a significant (*p* < 0.05) increase (9.6 ± 0.3 mm and 8.6 ± 0.2 mm). As shown in Figure 4D, most of the T4P-related mutants showed a significant decrease in swarming motility (*p* < 0.05), while R(PJD61) and R(PS3-2) showed a significant (*p* < 0.05) improvement (9.9 ± 0.2 mm and 12.9 ± 1.1 mm).

When T4P-mediated twitching motility was tested, significant decreases in twitching motility (*p* < 0.05) were observed among the T4P-, OMP-, and EPS-related mutants (Figure 4F). Among the mutants with large-fragment deletions, R(PS6-S) showed a significant (*p* < 0.05) decrease in twitching motility (10.4 ± 1.2 mm) compared to the wild-type PAO1 (13.3 ± 0.9 mm), but R(PSJ-22-5) and R(PSJ-22-5D) showed a significant (*p* > 0.05) increase (14.7 ± 1.0 mm and 14.7 ± 0.3 mm) (Figure 4E). However, R(PSJ-17-1-2) and R(PSJ-17-5D) showed completely opposite changes in twitching motility (Figure 4E, 11.4 ± 0.5 mm and 18.2 ± 0.7 mm), which might be explained by their different mutation sites in the same gene. The other LPS-related mutant strains showed no significant difference in twitching motility compared to the wild-type PAO1 (*p* > 0.05).

### 2.3. Biofilm Formation

**Figure 5:**
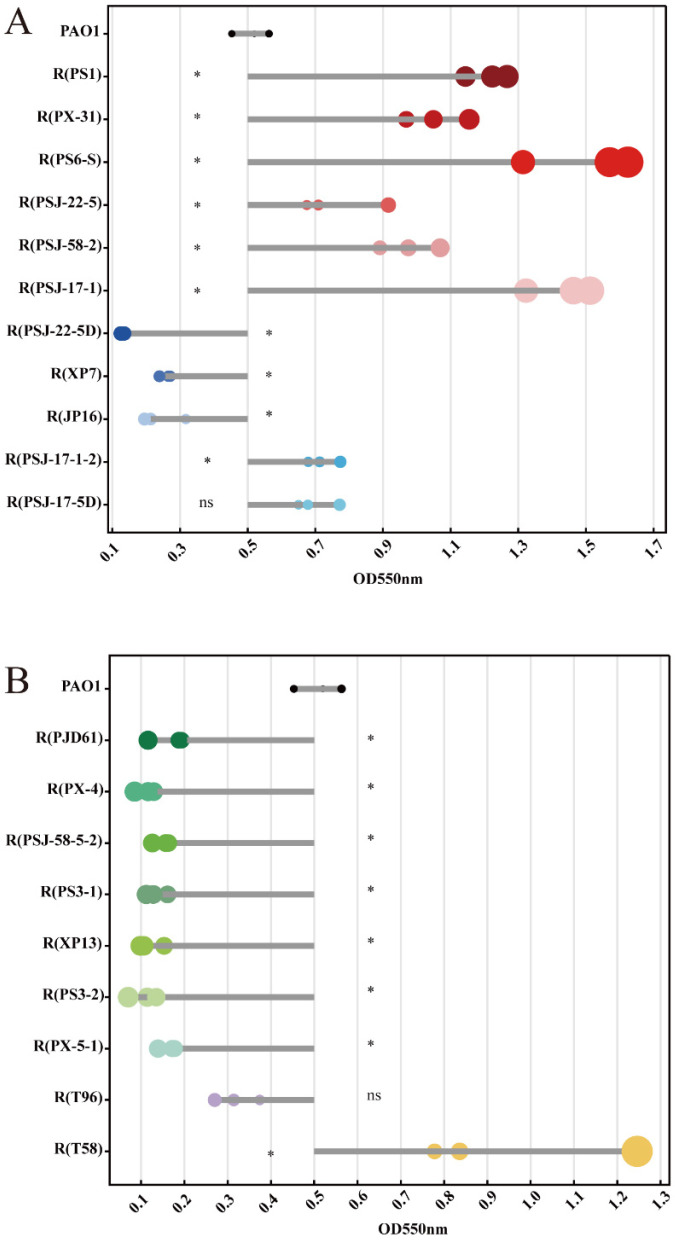
Biofilm formation abilities of PAO1 and its mutants, expressed as OD_550_ values using the crystal violet staining method. The size of the dot represents the absolute value of the OD_550_ value difference with PAO1. (**A**) LPS-related mutants. Red dots: large-fragment deletion mutation with brown phenotype. Blue dots: other LPS-related mutants. (**B**) T4P-related and other related mutants. Green dots: T4P-related mutants. Purple dot: OMP-related mutant. Yellow dot: EPS-related mutant. All data were statistically analyzed to evaluate the difference between the mutants and wild-type PAO1. Symbol * indicates the sample is significantly different (*p* < 0.05) from the control wild-type PAO1, and “ns” means no significance.

Among the LPS-related mutant strains (Figure 5A), all large-fragment deletion mutations with the brown phenotype characterized by red dots were observed to have significantly increased biofilm formation ability (*p* < 0.05), whereas the O-antigen-related gene *wzzB* mutant strains showed a significant decrease (*p* < 0.05). R(PSJ-17-1-2) and R(PSJ-17-5D), with different mutation sites in the glycosyltransferase gene, both showed enhanced biofilm formation ability, and R(PSJ-17-1-2) was significantly different compared to the wild-type PAO1 (*p* < 0.05). Since biofilm formation ability can be influenced by T4P-mediated twitching motility, the T4P-related mutant strains all showed a significant reduction in biofilm formation ability (*p* < 0.05), and this was consistent with the twitching motility assay (Figure 5B). The OMP mutant strain R(T96) showed no significant difference from the wild-type PAO1 (*p* > 0.05). The EPS-related mutant strain R(T58) showed a significant increase in biofilm formation ability (*p* < 0.05).

**Figure 6:**
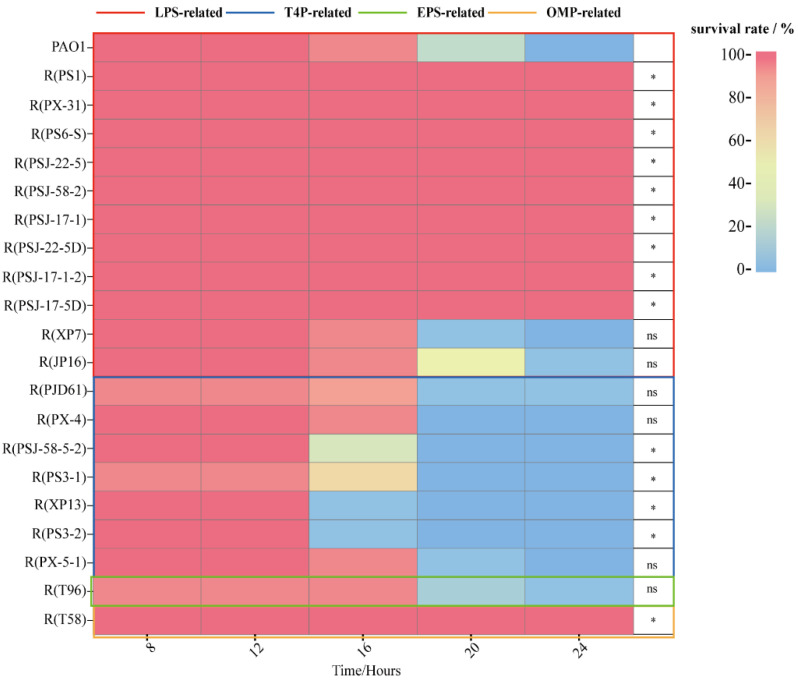
Survival rates in *G. mellonella* larvae after challenge with PAO1 or its mutants. Symbol * indicates the sample is significantly different (*p* < 0.05) from the control wild-type PAO1, and “ns” means no significance.

*Galleria mellonella* infection models. To compare the virulence between the phage-resistant strains and wild-type PAO1, we employed a *Galleria mellonella* (*G. mellonella*) larvae model to monitor the survival of larvae challenged with different strains [19]. As shown in Figure 6 and Figure S1, the larvae treated with the wild-type PAO1 were all dead after 24 h. However, the larvae of the other treatment groups, which were challenged with mutants carrying LPS-related mutations (large-fragment deletion and glycosyltransferase mutations) and the EPS-related mutation, all survival after 24 h. The groups treated with other LPS-related mutants, including R(XP7) and R(JP16), showed no significantly different survival rates compared with those treated with the wild-type PAO1 (*p* < 0.05). These results indicate that selective pressure had a great impact on reducing the virulence of most LPS-related mutant strains and the EPS-related mutant R(T58). In addition, the virulence of the T4P-related mutants was also investigated since motility has an indirect association with bacterial virulence. Motility is characterized by the ability to translocate to preferred hosts, the efficiency of nutrient acquisition, and access to optimal colonization sites within hosts [20]. The *G. mellonella* larvae virulence model demonstrated heterogeneity in the groups treated with the T4P-related mutants. Compared with the wild-type PAO1, the 24 h survival rates of the groups treated with R(PX-5-1), R(PX-4) and R(PJD61) showed no significant differences (*p* > 0.05). It is worth noting that the 24 h survival rates of the groups treated with R(PSJ-58-5-2), R(PS3-1), R(XP13), and R(PS3-2) showed a significant decrease (*p* < 0.05).

Antibiotic susceptibility tests. After analyzing the mutation results, we found that the large-fragment deletion strains lost antibiotic resistance-associated genes, such as *mexXY*, which may trigger increased antibiotic susceptibility. We determined the minimum inhibitory concentrations (MICs) of the wild-type PAO1 and fragment deletion strains against gentamicin, amikacin, levofloxacin, and ofloxacin, as described by Masuda et al. and Nakamura et al. [21,22]. As shown in Table 1, all large-fragment deletion strains showed a 16-fold increase in susceptibility to gentamicin and a 4- or 8-fold increase in susceptibility to amikacin. Unlike the two antibiotics of the aminoglycoside class, only R(PS1) and R(PX-31) showed twice the susceptibility to levofloxacin as compared to the wild-type PAO1; the rest of the strains did not show increased susceptibility to levofloxacin or ofloxacin.

## 3. Discussion

Phage therapy is becoming an antibiotic alternative due to the increasing prevalence of multidrug-resistant bacteria [23]. The emergence of phage-resistant bacteria is typically unfavorable for phage therapy [24], because bacteria cannot be killed directly by phages. However, a strategy called “phage steering” is based on the emergence of phage-resistant bacteria because the development of phage resistance is often accompanied by fitness costs [17]. We can exploit such phenomena by applying appropriate selective pressure to steer the evolution of phage-resistant bacterial strains in directions that are beneficial for treatment. In this study, 20 isolated phages were used to exert selective pressure on the *P. aeruginosa* strain PAO1 to obtain corresponding phage-resistant strains. Sequencing and comparative genomics were used to analyze the phage-resistant mutants with mutations in receptor-related genes, and various aspects of these resistant strains were evaluated, including growth, biofilm formation, motility, virulence, and antibiotic susceptibility.

This research indicates that bacteria became phage-resistant at high fitness costs. Most importantly, for phage steering, this study could help to determine whether the physiological changes caused by phage resistance are positive or negative for treatment. Based on these results, better phages or phage cocktails could be selected to guide the bacterial population toward a beneficial treatment direction.

LPS-related mutants with large-fragment deletions (in the *galU*-containing region), insertion, or SNP in the bacterial genome displayed varied bacterial fitness, because these genes are involved in biosynthesis, transcriptional regulation, and nutrient transport. The loss of *hmgA* results in the accumulation of the brown pigment pyomelanin, so mutants with large-fragment deletions including the *hmgA* gene often show a brown phenotype [22].

Different phage-resistant strains showed different levels of fitness costs in growth, including a prolonged lag phase, reduced maximum optical density (OD), and diminished growth rate, which were influenced by the selective pressure exerted by the phages. In growth assays, all mutants with large-fragment deletions showed a severe growth impairment, suggesting that growth is one of the adaptive trade-offs. On the one hand, the size of the deleted fragments on the genome affects the bacterial growth to different extents. For example, R(PSJ-22-5D) showed a minimum OD reduction among all the mutants with large-fragment deletions. On the other hand, mutations at different sites of the same gene also have different effects on bacterial growth, such as in the cases of the glycosyltransferase-gene-related mutants R(PSJ-17-1-2) and R(PSJ-17-5D). However, not all LPS-related mutations caused severe impairment of bacterial growth, which might be explained by different contributions of genes to fitness, like O-antigen-related mutants R(XP7) and R(JP16). Overall, the reduced fitness of bacteria caused by LPS-related mutations is a valid direction for phage steering to be applied in clinical therapy.

Swimming, swarming, and twitching are three classical types of motilities in *P. aeruginosa* [25], and they are crucial for reaching preferred infection sites, promoting biofilm formation, and adhering to surfaces or host cells [26]. Changes in bacterial motility in phage-resistant mutants can be utilized in phage therapy. LPS-related mutations can affect bacterial motility in different ways [27,28]. In motility assays, the large-fragment deletion mutants showed an enhancement in swimming motility and a reduction in swarming motility, which are both mediated by flagella. Similar phenomena have been reported indicating that genomic deletions in *P. aeruginosa* lead to defects in types of swarming and swimming motilities, but rather than causing defects in flagellar synthesis or function, cell hydrophobicity increases, resulting in stronger cell-to-cell binding [28]. In addition, O-antigens may mediate surface translocation by acting as surfactants or increasing cell surface “wettability” [29]. So, a fragment deletion in the genome and the mutations associated with the O-antigen might induce a decrease in the swarming ability of mutants. For phage steering, the screening of LPS-targeted selective pressure can indeed reduce the motility in bacterial populations and further reduce the migration ability of bacteria in hosts.

Biofilm formation is related to virulence, antibiotic resistance, and other factors [30], which is advantageous for bacteria to adapt to environments. Biofilm formation can be regulated by many factors. Flagellar-mediated swimming and T4P-mediated twitching have an important association with the early stages of biofilm formation, and EPS also plays a crucial role [31,32,33]. LPS, as a highly dynamic structure, plays an important role in biofilm formation [34]. In the early stages of biofilm development, changes in the LPS core capped by the O-specific antigen (OSA) can affect biofilm structure by altering adhesion, cohesion, and viscoelasticity, and the common polysaccharide antigen (CPA) plays a stronger role than OSA in forming a strong biofilm [35]. In this biofilm assessment assay, the OSA mutant strains showed a significant decrease in biofilm formation (*p* < 0.05), confirming the positive effects in biofilm formation regulated by the *wzzB* and *wzy* genes. All large-fragment deletion strains exhibited significant increases in biofilm formation (*p* < 0.05), except for R(PSJ-22-5D). This difference may be related to the phenotypes associated with the deletions of *hmgA* and other genes. Overall, these results support the notion that selecting O-antigen-targeting phages to apply selective pressure can weaken the formation of bacterial biofilms.

To improve therapeutic efficacy, screening for phage-resistant mutants with reduced virulence is one application of phage steering [35]. LPS is an important virulence factor of *P. aeruginosa* [36] and one of the most common receptors for phages. In our *G. mellonella* virulence model, most of the LPS-related mutants, including the large-fragment deletion strains and the mutants with PTC in glycosyltransferases, showed significant decreases (*p* < 0.05) in virulence when compared to the wild-type PAO1, suggesting that they are applicable in phage steering, as described by Lau et al. [35]. Additionally, Van Nieuwenhuyse et al. [37] and Li et al. [38] showed that phage steering based on decreased virulence was well used in clinical therapy.

Mutations in antibiotic resistance-associated genes caused by phage selective pressure allow bacteria to make trade-offs between phage resistance and antibiotic resistance [39]. Ricci et al. [40] and Chan et al. [41] took advantage of phage resistance to re-sensitize pathogens to antibiotics when the phage receptor was also the efflux pump for the antibiotic and proposed phage–antibiotic combination therapy based on their findings. In clinical therapy, Bao et al. [42] employed the combination of sulfamethoxazole–trimethoprim with a phage cocktail to successfully cure a patient with a recurrent urinary tract infection. Herein, we investigated the effects of the pressure of different phages on the antibiotic susceptibility of *P. aeruginosa* and observed that mutants with antibiotic resistance-associated gene deletions had increased antibiotic susceptibility. Firstly, we identified several antibiotic resistance-associated genes, such as the OprD family porin, multidrug efflux RND transporter periplasmic adaptor subunit MexX, multidrug efflux RND transporter permease subunit MexY, multidrug efflux SMR transporter, and TolC family protein, in the LPS-associated mutants with large-fragment deletions. Then, four antibiotics associated with *mexXY* and other deleted genes [43] were used to evaluate the antibiotic susceptibility of these mutants. As expected, these strains exhibited increased antibiotic susceptibility, in which the sensitivity to gentamicin and amikacin was increased by 16/4 or 8-fold. Therefore, increased antibiotic susceptibility is one of the favorable outcomes in phage-resistant mutants associated with LPS-related genes, and this positive feedback could be applied for phage–antibiotic combination therapy.

Among the 20 phage-resistant PAO1 strains, T4P-related gene mutations were present in 7 cases. The mutations directly associated with T4P included the lower periplasmic ring genes *pilN* and *pilO*, cytoplasmic ring gene *pilM* [44], fiber-associated pilin-like protein gene *pilX* [45], and pilus-associated adhesin and anti-retraction protein gene *pilY1* [46]. Two other genes indirectly related to T4P included the two-component transcriptional regulator gene *pilR* [47] and the GspE/PulE family ATPase, which provides power to the T4P system [48]. In the other two phage-resistant strains, one was related to TonB-dependent receptors as outer membrane proteins, which participate in the uptake of essential nutrients, including iron [49], and the other one was related to EPS, namely, the anti-sigma factor gene *mucA*, which inhibits the sigma factor gene *algU*, causing R(T58) to exhibit a mucoid phenotype [50].

Compared to the high fitness cost in bacterial growth caused by LPS mutations, the strains with mutations related to T4P and OMP did not show significant differences in comparison with the wild-type PAO1 (*p* < 0.05). However, R(T58) with EPS-related mutations showed the highest fitness cost among all phage-resistant strains. The explanation might be that the mutation of *mucA* leads to the overproduction of alginate and then inevitably affects the growth metabolism of the bacteria [50].

All T4P mutant strains showed a significant decrease in twitching motility, but the effects of T4P-related mutations on other motilities showed considerable heterogeneity. For example, the *pilSR* deletion mutant had a defect in swimming motility [47], and the *pilR* and *pilA* mutants were unable to swarm [51]. These findings indicate that flagella-mediated swimming and swarming motilities are also affected by T4P-related genes. For the *mucA* mutant R(T58) with a mucoid phenotype, its resulting overexpression of *estA* led to a significant decrease in twitching motility (*p* < 0.05), although it played an important role in enhancing swimming and swarming [52]. The motility variations in the TonB-dependent receptor mutant might be caused by the indirect effect of the mediate energy-dependent uptake of molecule changes in flagella and T4P [53]. Consequently, using T4P-targeted phages to apply selective pressure in phage steering can effectively make the population evolve toward a twitching-deficient phenotype, thereby affecting a series of physiological characteristics mediated by T4P and twitching motility.

The T4P-related mutants all exhibited a decreased biofilm formation capacity because T4P is crucial for the initial phases of biofilm formation [54]. However, the *mucA* mutant showed a greatly enhanced ability to form biofilms due to the extracellular mucoid effect on biofilm formation [52]. This indicates that phages using T4P as bacterial receptors could be a good choice for phage steering to weaken the biofilm formation ability of bacterial populations.

In addition to mediating twitching motility, T4P also acts as a sensor for regulating surface-induced gene expression and pathogenicity [55,56]. In the *G. mellonella* larvae infection model, phage-resistant strains with *pilX*, *pilM*, *pilR*, and *pilY1* mutations showed enhanced virulence. Similar phenomena have been reported in the variant libraries of *P. aeruginosa* PAO1 with resistance to phages PP7 and E79, and many genes related to phage resistance and *Pseudomonas* virulence were found to be upregulated in these mutants [57]. The mucoid phenotypic strain R(T58) selected by phages had a decrease in virulence that was similar to the mucoid phenotypes isolated from cystic fibrosis patients [58]. However, we still need to consider other effects caused by excessive alginate as the main pathogenic determinant during chronic respiratory tract infection [59]. Although the evolutionary direction of bacterial populations under phage selection pressure do not always evolve in a beneficial direction for treatment, the study of phage-resistant strains can help us choose appropriate phage-steering strategies and reduce the emergence of hard-to-treat phage-resistant bacteria populations.

In this work, we imposed phages to *P. aeruginosa* PAO1 and obtained 20 mutants with mutations related to LPS, T4P, OMP, and EPS. The growth, motility, biofilm formation, virulence, and antibiotic sensitivity of these strains were systematically evaluated. Due to the complexity of bacterial infections and the diversity of phage types, phage resistance-induced changes in bacterial physiological characteristics are heterogeneous. On the one hand, employing LPS-targeted phages might significantly reduce the virulence of pathogens or make them re-sensitize to antibiotics, and both outcomes are favorable for phage therapy or phage–antibiotic combination therapy. On the other hand, T4P-targeted phages can be used to screen for defective populations in twitching motility and biofilm formation, which are more effective in clearing bacteria from the environment. However, our findings also revealed that mutants resistant to one single phage might evolve toward unfavorable therapeutic directions, triggering the usage of phage cocktails to maximize the profits of phage steering in the future. In conclusion, our work provides a basis for understanding phage-resistant mutants and for informing the rational selection of phages or phage cocktails to respond to different needs for clinical treatments. As the adaptive trade-off mechanisms of phage resistance are further investigated, phage steering will be increasingly used as an effective strategy for clinical treatment.

## 4. Materials and Methods

Phages and phage-resistant strains. *P. aeruginosa* PAO1 was used as a host to isolate phages from sewage samples of different hospitals in China in our previous work. Twenty phages infecting *P. aeruginosa* PAO1 were obtained, and their genome information is shown in Table S1.

Phage-resistant strains were prepared via the double-layer method. Briefly, 200 μL of phage solution (10^8^ PFU/mL) and 100 μL of overnight PAO1 culture were added to 5 mL of LB soft agar and incubated at 37 °C overnight. After overnight growth, the phage-resistant bacteria were picked up from the overlayed plates, and they were inoculated into 5 mL of LB broth medium and incubated at 37 °C with shaking for 8 h.

Adsorption assays of phages. Double-layer plates containing 200 μL overnight PAO1 culture in soft agar were prepared for adsorption assays, as described by Piel et al. [60]. Phages (10^6^ PFU/mL) were mixed with PAO1 and phage-resistant strains (10^8^ CFU/mL) at an MOI of 0.01 at room temperature for 15 min. A control group was treated by adding phosphate-buffered saline (PBS, Biosharp, Hefei, China). Then, the mixture was centrifuged at 12,000× *g* for 10 min to discard cells adsorbed with phages. Afterward, the supernatant was 10-fold serially diluted, and 2 μL of each dilution was dropped onto the soft agar to determine the remaining free phage particles. The experiments were performed at least twice. The adsorption capacity of phage-resistant strains to phages was estimated by comparing the number of remaining free phage particles with that of the wild-type PAO1 strain. For instance, if the free phage particles were more than the wild-type PAO1, the phage-resistant phenotype could be confirmed through the impaired adsorption performance.

Genomic DNA extraction, sequencing, and a comparative genomic analysis of phage-resistant strains. The genomes of all phage-resistant strains with impaired adsorption were extracted using a Bacterial Genomic DNA Extraction Kit (Solarbio, Beijing, China). The sequencing of phage-resistant bacteria was carried out by Novogene Co., Ltd. (Qingdao, China accessed on 7 December 2022), and the sequencing platform was Illumina Novaseq 6000 (Paired-end 150 bp). The quality of the raw sequencing data was analyzed using FastQC v0.11.5, and low-quality reads and adapter regions were filtered using Trimmomatic 0.36 with default parameters [61]. The clean data were assembled using SPAdes v3.13.0 [62], and mutations in resistant strains were analyzed using breseq v0.37.1 [63].

The growth curves of PAO1 and its phage-resistant strains. To evaluate the growth rates of PAO1 and its phage-resistant strains, each strain was introduced into the wells of a 96-well plate (Corning-Costar, New York, NY, USA) containing 200 μL LB liquid medium to reach a final concentration of 10^6^ CFU/mL. Subsequently, the plate was incubated at 37 °C for 24 h. A control group was established without adding any bacterial strains. The OD_600_ of the 96-well plate was measured every hour using Thermo Scientific™ Multiskan™ FC (Thermo Fisher Scientific, Shanghai, China). The growth curves of PAO1 and its phage-resistant strains were generated based on the OD_600,_ and each growth curve assay was performed in triplicate.

Motility assays. Swimming and swarming assays were carried out with 0.3% LB agar plates and 0.5% LB agar plates, respectively, as described by Xuan et al. [64], with a minor revision. A single colony of PAO1 or its phage-resistant mutants were inoculated into swimming or swarming plates at 1/2 depth, respectively, and then the swimming or swarming zones were recorded after incubating at 37 °C for 24 h. To conduct the twitching assay, PAO1 and its phage-resistant mutants were stab-inoculated to the bottom of the 1% LB agar layer with a pipette tip. After incubation at 37 °C for 48 h, the agar was carefully removed, and the Petri dish was stained with 0.1% crystal violet ammonium oxalate solution (Solarbio, Beijing, China) for 30 min. Then, the twitching mobility zone was measured after excess dye was washed away. All plates were photographed using Tanon 4600SF (Tanon, Shanghai, China), and the diameters of the mobility zones were measured using Nano Measurer 1.2.5 [65].

Biofilm formation assays. The crystal violet staining method was used to evaluate the biofilm formation of bacterial strains [64]. Briefly, PAO1 and its mutants were inoculated in a 96-well plate containing 150 μL LB broth and incubated at 37 °C for 24 h. Subsequently, non-adherent bacterial cells were removed, and the wells were washed thrice with 200 μL of PBS. To stain and assess the biofilm, 200 μL of 1% crystal violet ammonium oxalate solution (Solarbio, Beijing, China) was added to each well and allowed to incubate at room temperature for 30 min. The excess crystal violet solution was washed away with PBS, and the plate was dried at room temperature for 30 min. Afterward, 200 μL of 30% acetic acid solution was added to each well and incubated at room temperature with 100 rpm for 20 min. Each experiment was performed in triplicate. The OD_550_ was measured using a Thermo Scientific™ Multiskan™ FC to quantitatively determine the biofilm formation.

Antibiotic susceptibility tests. The MICs of each antibiotic against *P. aeruginosa* PAO1 and the phage-resistant strains with large-fragment deletions were determined in 96-well microplates via the double dilution of each antibiotic in CAMHB (Cation-Adjusted Mueller Hinton II Broth, Solarbio, Beijing, China). Then, 1.0 × 10^6^ CFU/mL of each strain was inoculated into each well and incubated at 37 °C for 24 h, and PBS was inoculated as a control in each assay. Each experiment was conducted in triplicate. MIC was defined as the concentration present in the first optically clear well after incubation.

*G. mellonella* larvae infection models. The larvae of *G. mellonella* were purchased from Huiyude Biotech Company (Tianjin, China), and the *G. mellonella* infection assay was based on the method described by Hill et al., with modifications [66]. Briefly, 6 μL of 10^5^ CFU/mL bacterial strains was injected into the hindmost proleg of each *G. mellonella* larva. The experiment was conducted with 10 larvae in each group. The infected larvae were incubated at 37 °C, and their health index scoring for live/dead was examined at 8, 12, 16, 20, and 24 h. The negative control group comprised 10 larvae that were injected with equivalent PBS.

Statistical analysis. All data are expressed as means ± SD (standard deviation), and the index in different assays was statistically analyzed using different methods to evaluate the difference between the wild-type PAO1 and mutants (denoted as *p* < 0.05). An Analysis of Variance (ANOVA) was used to analyze the difference in the growth, motility, and biofilm assays, and the log-rank test was used to analyze the survival rates in the *G. mellonella* larvae infection model. A statistical analysis was carried out using GraphPad Prism v.8.0.2 software. *p*-value < 0.05 was considered statistically significant.

## Figures and Tables

**Table 1:** Minimum inhibitory concentrations (MICs) of antibiotics against PAO1 and large-fragment deletion mutants.

Name	Aminoglycoside		Quinolone	
	Gentamicin MIC (mg/L)	Amikacin MIC (mg/L)	Levofloxacin MIC (mg/L)	Ofloxacin MIC (mg/L)
PAO1	4	8	0.5	1
R(PS1)	0.25	1	0.25	1
R(PX-31)	0.25	1	0.25	1
R(PS6-S)	0.25	2	0.5	1
R(PSJ-22-5)	0.25	2	0.5	1
R(PSJ-58-2)	0.25	2	0.5	1
R(PSJ-17-1)	0.25	1	0.5	1
R(PSJ-22-5D)	0.25	2	0.5	1

## Data Availability

All data generated for this study are included in the article and Supplementary Materials. The genome sequence of wild-type PAO1 and mutants were deposited at the National Centre for Biotechnology Information (NCBI), under the BioProject accession number PRJNA963233.

## Supplementary Materials

The supporting information can be downloaded at https://www.mdpi.com/article/10.3390/ijms242115594/s1.
