# Supplementary material for: Genetic and Phenotypic Analysis of Phage-Resistant Mutant Fitness Triggered by Phage–Host Interactions
Source: Int J Mol Sci. 2023 Oct 26;24(21):15594. doi: 10.3390/ijms242115594 (PMC10648725; doi:10.3390/ijms242115594)
Supplement: Supplementary file 1 [file ijms-24-15594-s001.zip › ijms-2662574-supplementary.pdf]

Table S1. Genetic changes of phage-resistant strains *via* comparative genomic analysis.

Mutant Name	Phage Name	Genome Size of Phage	Position	Mutation	Annotation	Description
R(PS1)	v-PS1	93543 bp	3,074,784	Δ403,605 bp	Deletion	355 genes
R(PX-31)	v-PX-31	66534 bp	3,077,575	Δ403,180 bp	Deletion	356 genes
R(PS6-S)	v-PS6-S	93543 bp	3,082,481	Δ305,744 bp	Deletion	281 genes
R(PSJ-22-5)	v-PSJ-22-5	66571 bp	3,082,920	Δ277,257 bp	Deletion	251 genes
R(PSJ-58-2)	v-PSJ-58-2	66296 bp	3,204,984	Δ264,714 bp	Deletion	226 genes
R(PSJ-17-1)	v-PSJ-17-1	66301 bp	3,278,588	Δ196,584 bp	Deletion	172 genes
R(PSJ-22-5D)	v-PSJ-22-5D	92962 bp	3,274,638	Δ29,905 bp	Deletion	30 genes
R(XP7)	v-XP7	66487 bp	4,486,087	A→T	SNP	LPS O-antigen chain length determinant protein WzzB
R(JP16)	v-JP16	72406 bp	1,976,280	$(A)_{7→8}$	Insertion	O-antigen polymerase Wzy
R(PSJ-17-1-2)	v-PSJ-17-1-2	66300 bp	5,629,549	G→T	PTC	glycosyltransferase
R(PSJ-17-5D)	v-PSJ-17-5D	66279 bp	5,628,939	C→A	PTC	glycosyltransferase
R(PJD61)	v-PJD61	43175 bp	5,689,125	+G	Insertion	type 4a pilus biogenesis protein PilO
R(PX-4)	v-PX-4	43010 bp	5,689,512	G→A	SNP	type 4a pilus biogenesis protein PilN
R(PSJ-58-5-2)	v-PSJ-58-5-2	72406 bp	5,100,172	C→T	PTC	type 4a pilus biogenesis protein PilY1

R(PS3-1)	v-PS3-1	43010 bp	5,099,533	Δ16 bp	Deletion	type 4a pilus minor pilin PilX
R(XP13)	v-XP13	61076 bp	5,690,050	Δ12 bp	Deletion	type IV pilus assembly protein PilM
R(PS3-2)	v-PS3-2	43010 bp	5,095,174	Δ162 bp	Deletion	two-component system response regulator PilR
R(PX-5-1)	v-PX-5-1	43010 bp	5,875,331	Δ226 bp	Deletion	inorganic triphosphatase, GspE/PulE family protein
R(T96)	v-T96	73576 bp	2,248,033	Δ1 bp	Deletion	TonB-dependent receptor
R(T58)	v-T58	93056 bp	4,682,915	+CTCC	Insertion	anti-sigma factor MucA

Table S2. Diameter of the growth zone for three motility assays.

Mutant Name	Swimming (mm)	Swarming (mm)	Twitching (mm)
PAO1	13.008±0.184	8.296±0.114	13.300±0.862
R(PS1)	15.805±0.366	6.737±0.232	13.957±0.767
R(PX-31)	17.136±0.194	6.590±0.190	12.707±0.757
R(PS6-S)	16.235±0.312	6.282±0.186	10.415±1.212
R(PSJ-22-5)	16.476±0.353	6.946±0.277	14.689±1.026
R(PSJ-58-2)	15.742±0.457	6.692±0.157	13.530±1.121
R(PSJ-17-1)	17.055±0.266	5.721±0.199	13.716±1.862
R(PSJ-22-5D)	16.497±0.324	6.782±0.314	14.651±0.323
R(XP7)	14.115±0.286	8.648±0.229	14.521±0.737
R(JP16)	10.864±0.421	7.116±0.277	13.284±1.012
R(PSJ-17-1-2)	13.697±0.416	9.641±0.258	11.387±0.471
R(PSJ-17-5D)	12.197±0.153	4.877±0.209	18.157±0.716
R(PJD61)	14.234±0.227	9.906±0.179	4.551±0.618
R(PX-4)	12.888±0.307	5.853±0.532	8.155±0.363
R(PSJ-58-5-2)	15.219±0.532	8.760±0.266	4.594±0.174
R(PS3-1)	14.668±0.190	5.086±0.318	5.053±0.943
R(XP13)	11.072±0.706	7.142±0.219	7.088±0.509

R(PS3-2)	14.912±0.200	12.891±1.127	4.477±0.274
R(PX-5-1)	15.857±0.248	7.648±0.162	5.959±0.444
R(T96)	14.810±0.229	5.598±0.185	7.540±1.318
R(T58)	18.354±0.788	10.611±0.274	8.437±0.891

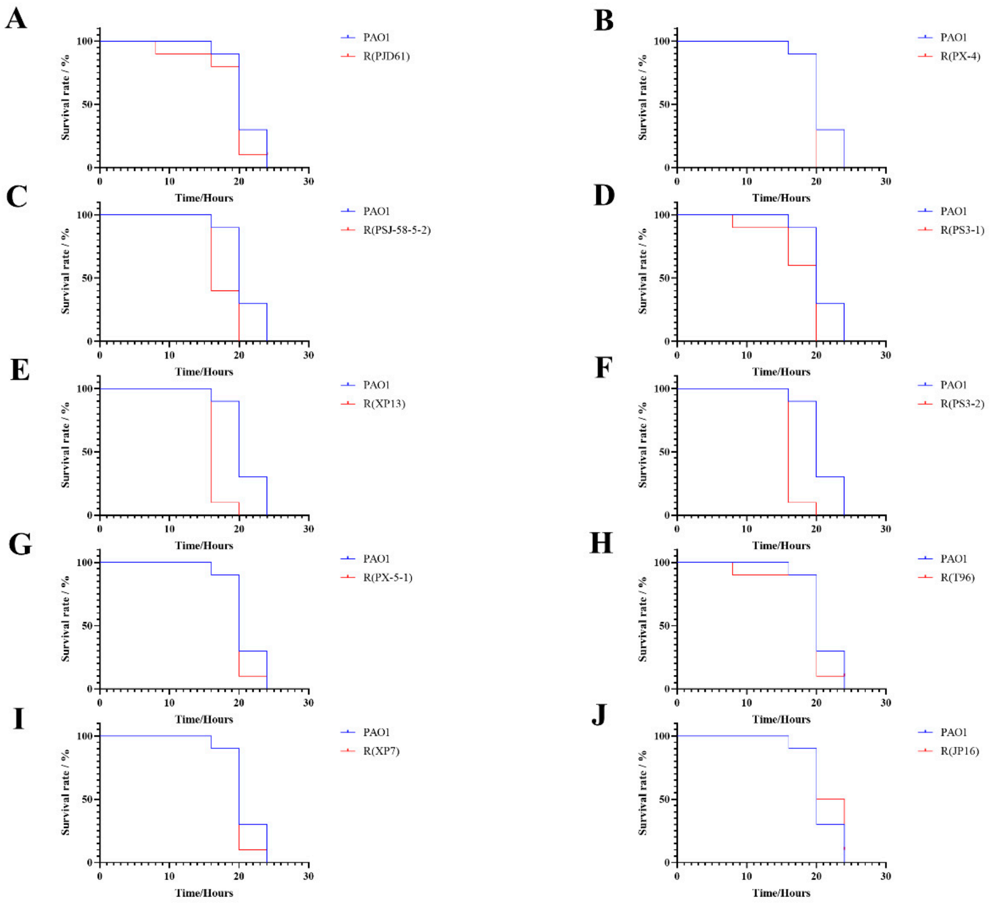

Figure S1. Survival curves of phage-resistant mutants (mutants with 100% survival rates were not shown).
